# The Association of LINE-1 Hypomethylation with Age and Centromere Positive Micronuclei in Human Lymphocytes

**DOI:** 10.1371/journal.pone.0133909

**Published:** 2015-07-21

**Authors:** Yoon Hee Cho, Hae Dong Woo, Yoonhee Jang, Virginia Porter, Sonja Christensen, Raymond F. Hamilton, Hai Won Chung

**Affiliations:** 1 Center for Environmental Health Sciences, Department of Biomedical & Pharmaceutical Sciences, The University of Montana, Missoula, Montana, United States of America; 2 Molecular Epidemiology Branch, Division of Cancer Epidemiology and Prevention, Research Institute, National Cancer Center, Goyang-si, Gyeonggi-do, Korea; 3 Department of Psychology, The University of Montana, Missoula, Montana, United States of America; 4 Department of Molecular Epidemiology, School of Public Health, Seoul National University, Gwanak-gu, Seoul, Korea; CEA - Institut de Genomique, FRANCE

## Abstract

Global hypomethylation in white blood cell (WBC) DNA has recently been proposed as a potential biomarker for determining cancer risk through genomic instability. However, the amplitude of the changes associated with age and the impacts of environmental factors on DNA methylation are unclear. In this study, we investigated the association of genomic hypomethylation with age, cigarette use, drinking status and the presence of centromere positive micronuclei (MNC+)—a biomarker for age-dependent genomic instability. Genomic hypomethylation of the repetitive element LINE-1 was measured in WBC DNA from 32 healthy male volunteers using the pyrosequencing assay. We also measured MNC+ with the micronucleus-centromere assay using a pan-centromeric probe. Possibly due to the small sample size and resulting low statistical power, smoking and drinking status had no significant effect on LINE-1 hypomethylation or the occurrence of MNC+. Consequently, we did not include them in further analyses. In contrast, LINE-1 hypomethylation and age significantly predicted MNC+; therefore, we examined whether LINE-1 hypomethylation plays a role in MNC+ formation by age, since genomic hypomethylation is associated with genomic instability. However, LINE-1 hypomethylation did not significantly mediate the effect of age on MNC+. Our data indicate that the repetitive element LINE-1 is demethylated with age and increasing MNC+ frequency, but additional studies are needed to fully understand the relation between genomic DNA hypomethylation, age and genomic instability.

## Introduction

DNA methylation, a well-defined epigenetic mechanism, is mitotically inheritable while remaining modifiable through environmental interactions. It also plays an important role in causing chronic diseases by silencing genes through hypermethylation or activating genes through hypomethylation [1–4]. Genomic DNA hypomethylation resulting from demethylation in transposable repetitive elements is associated with genomic instability and is an independent predictor of cancer risk [5]. Numerous epidemiological studies have shown that demographics (gender, race and age) [6–8], environmental exposures (benzene, lead, persistent organic pollutants and particulate matter) [9–12], and life styles (diet, smoking, alcohol, BMI and physical activities) [13–16] are potential risk factors for increasing disease risk correlated with global DNA methylation.

In particular, growing evidence suggests that changes in global DNA methylation occur over time [7, 8, 17]. Several studies have shown that genomic DNA hypomethylation is associated with cellular senescence and aging within *in vitro* and animal models [18–20]. In humans, global DNA hypomethylation has been found in a variety of age-related diseases [21–24] and observed with age [8]. However, the results are far from consistent, and there is a significant need for studies aimed at quantifying the degree of hypomethylation that occurs with age and the status following certain exposures.

Micronuclei (MN) in blood lymphocytes are a well-known cytogenetic biomarker for genomic instability induced by environmental exposures as well as aging [25, 26]. The age-effect on MN frequency was confirmed by data from the Human MicroNucleus Project with nearly 7,000 subjects [27]. The increase in MN frequency with age has been shown to be mainly due to increased MN containing centromeres (centromere-positive MN (MNC+)). Vral et al.[28] reported that a high percentage of spontaneous MN are also MNC+. Our previous study also showed that MNC+ frequency significantly increases with age, suggesting that the presence of MNC+ may serve as a valuable age-dependent biomarker for genomic instability [29]. However, the mechanisms underlying age-induced genomic instability and disease risk are not yet clear. Therefore, we examined whether LINE-1 hypomethylation plays a role in age-associated MNC+ formation, since there appears to be a relationship between genomic hypomethylation and instability.

In the present study, the micronucleus-centromere assay using a pancentromeric probe and methylation of the LINE-1 repetitive element by pyrosequencing was performed to determine the amplitude of global DNA methylation in white blood cell (WBC) DNA from 32 male volunteers in relation to their age, MNC+, smoking and drinking status.

## Materials and Methods

### Study population

The study population included 32 healthy male volunteers in Seoul, Korea. The volunteers were office workers who had never been occupationally exposed to medical irradiation, chemicals or mutagens. Information regarding smoking, drinking habits, medical history and drug intake were obtained via personal interviews. Participants did not have a personal medical history of cancer, genetic or other chronic disease and had no drug intake in the months prior to the study. Peripheral blood from each subject was drawn at the same time as the interview.

The local ethics committee approved the study protocol (Research Ethics Review Board of Korea Institute of Radiological and Medical Science) and written informed consents were obtained from each individual.

### Measurement of repetitive element, LINE-1 methylation

#### DNA preparation and bisulfite treatment

Genomic DNA was isolated from blood samples using the Wizard DNA Purification Kit (Promega, Madison, WI). 1 μg of DNA was bisulfite treated with the EZDNA Methylation Kit (Zymo Research, Irvine, CA) that converts nonmethylated cytosines to uracils while leaving methylated cytosines unmodified. The DNA was re-suspended in 20 μL of distilled water and stored at -20°C until further use.

#### Pyrosequencing assay

The methylation status of LINE-1 was measured by pyrosequencing. The primer sequences and PCR conditions have been previously described in detail [30, 31]. Briefly, PCR was carried out in a 25 μL reaction mix containing 50ng bisulfite-converted DNA, 1x Pyromark PCR Master Mix (Qiagen, Valencia, CA), 1x Coral Load Concentrate (Qiagen) and 0.2 uM forward and reverse primers, using the following PCR program: 95°C for 15 minutes, then 44 cycles of 95°C for 30 seconds followed by 56°C for 30 seconds and 72°C for 30 seconds, with a final extension at 72°C for 10 minutes. The biotinylated PCR products were purified and converted into single-strands to act as a template in the pyrosequencing reaction as recommended by the manufacturer of the Pyrosequencing Vacuum Prep Tool (Qiagen). Then, 0.3 nmol/L of pyrosequencing primer was annealed to the purified single-stranded PCR product and pyrosequencing was conducted on a PyroMark Q96 MD (Qiagen). We used non-CpG cytosine residues as internal controls to verify efficient sodium bisulfite DNA conversion and universal unmethylated and methylated DNAs (Zymo Research) were used as experimental controls. The intra- and inter-assay coefficients of variation were 0.7% and 1.4%, respectively.

### Measurement of MNC+

#### Cytokinesis-block micronucleus assay (CBMN)

1 ml of heparinized blood was mixed with 9 ml of culture medium (RPMI 1640; Gibco, Invitrogen Corporation, Carlsbad, CA) supplemented with 10% fetal bovine serum (Gibco), 100 units/ml penicillin (Gibco) and 1% phytohemagglutinin (Gibco). The cultures were incubated at 37°C in an atmosphere of 95% air and 5% CO_2_. Cytochalasin-B (4.0 μg /ml, Sigma, St. Louis, MO) was added after 44 hours from the start of the culture, followed by another incubation for 28 hours. The cells were collected and treated with 0.075M KCl hypotonic solution for 3 minutes and fixed in a mixture of methanol:acetic acid (3:1). The cells were smeared on pre-cleaned microscope slides and air-dried. The slides were stored at −20°C until their use for centromere labeling.

#### Interphase fluorescent in situ hybridization (FISH)

FISH was performed using a human pan-centromeric probe (Cambio, Cambridge, UK) directly labeled with fluorescein isothiocyanate (FITC) according to the manufacturer’s instructions. In brief, slides were pre-treated with proteinase K, then denatured in 70% (v/v) formamide/2x Saline-sodium citrate buffer (SSC) for 2 min (pH 7.3) at 72°C and subsequently dehydrated in graded 70, 85, and 100% (v/v) ethanol. The DNA probe was denatured at 85°C for 10 minutes then applied to the slides, which were then cover-slipped, sealed, and incubated in a humidified chamber. Following an overnight hybridization at 42°C, slides were washed twice in 50% formamide/2x SSC for 5 minutes at 37°C. Signals of the FITC hybridized probes were then amplified and counterstained with a FITC Amplification Kit (Cambio).

#### Slide scoring

The hybridized slides were randomized and coded blindly before examination. The presence of centromeres in the MN was observed with an epi-fluorescence microscope (Nikon, Tokyo, Japan), equipped with two filters for 4’,6’-diamidino-2-phenyloindole and FITC (Chroma Technology Corp., Brattleboro, VT, USA). For each slide, one reader scored 1000 binucleated (BN) cells according to criteria suggested by Fenech et al [26].

### Statistical analysis

Descriptive statistics were performed on data collected from the participants regarding age, smoking status, and alcohol intake (as self-reported data), as well as MNC+ and LINE-1 hypomethylation (experimental data). We subsequently performed inferential statistics. The effect of smoking status and alcohol intake on LINE-1 hypomethylation and MNC+ were tested considering that these were independent variables in a quasi-experimental design [32]. Further, to examine the relation between age, LINE-1 hypomethylation, and MNC+, a mediational model was constructed as a causal model (i.e., indicating paths between variables). Then, the four steps necessary for testing mediation were used [33–38] as follows: step 1 estimated the effect of age on MNC+ in a regression equation, step 2 estimated the effect of age on LINE-1 hypomethylation, step 3 examined the effect of age and LINE-1 hypomethylation on MNC+, and step 4 tested whether LINE-hypomethylation completely (or in part) mediates the path from age to MNC+; if LINE-1 hypomethylation is a perfect mediator, then the effect of age on MNC+ controlling for LINE-hypomethylation should be zero. Below, statistical significance was determined with an alpha of 0.05.

## Results

The general characteristics of the study participants are listed in Table 1. The participants were all male and their ages ranged from 21 to 57 years with a mean of 38.0 ±10.0 years. The mean MNC+ frequency obtained from the micronucleus-centromere assay was 5.3± 2.1‰ (range 1–8). The mean % of LINE-1 hypomethylation in WBC DNA from the participants was 74.4 ± 1.1 (range 71.8–77.5). Except for one participant who did not report whether he had a history of smoking, 9 participants (29%) were self-identified as being nonsmokers. Thirteen participants (41%) reported that they did not consume alcohol.

**Table 1:** Characteristics of the participants.

		Mean	S.D.	Range	Number of participants
Age (year)		38.0	10.0	21–57	-
MNC+^a^		5.3	2.1	1–8	-
LINE-1 hypomethylation (%)		74.4	1.1	71.8–77.5	-
Smoking ^b^	Current	-	-	-	7 (23%)
	Former	-	-	-	15 (48%)
	Never	-	-	-	9 (29%)
Drinking ^c^	Yes	-	-	-	19 (59%)
	No	-	-	-	13 (41%)

^a^ The frequency of MNC+ per 1000 BN cells

^b^ There was one missing value, and the total number of participants was 31.

^c^ Drinking status was classified as either a current alcohol drinker (yes) or not (no).

Table 2 shows mean values of MNC+ and LINE-1 hypomethylation for participants grouped according to their smoking and drinking status. There were no effects on LINE-1 hypomethylation and MNC+ due to smoking, *F*(2, 28) = 2.40, *p* = 0.11; similarly, there were no effects due to alcohol consumption, *t*(30) = 0.92, *p* = 0.36.

**Table 2:** Mean values of MNC+ and LINE-1 hypomethylation stratified by smoking and drinking status.

		MNC+^a^ (Mean (SD))	LINE-1 (Mean % (SD))
Smoking	Current	6.1 (1.5)	74.0 (1.1)
	Former	5.5 (2.2)	74.3 (1.1)
	Never	4.1 (1.8)	74.9 (1.2)
Drinking ^b^	Yes	5.3 (2.2)	74.6 (1.2)
	No	5.5 (1.8)	74.2 (1.0)

^a^ The frequency of MNC+ per 1000 BN cells

^b^ Drinking status was classified as either a current alcohol drinker (yes) or not (no).

**Fig 1:**
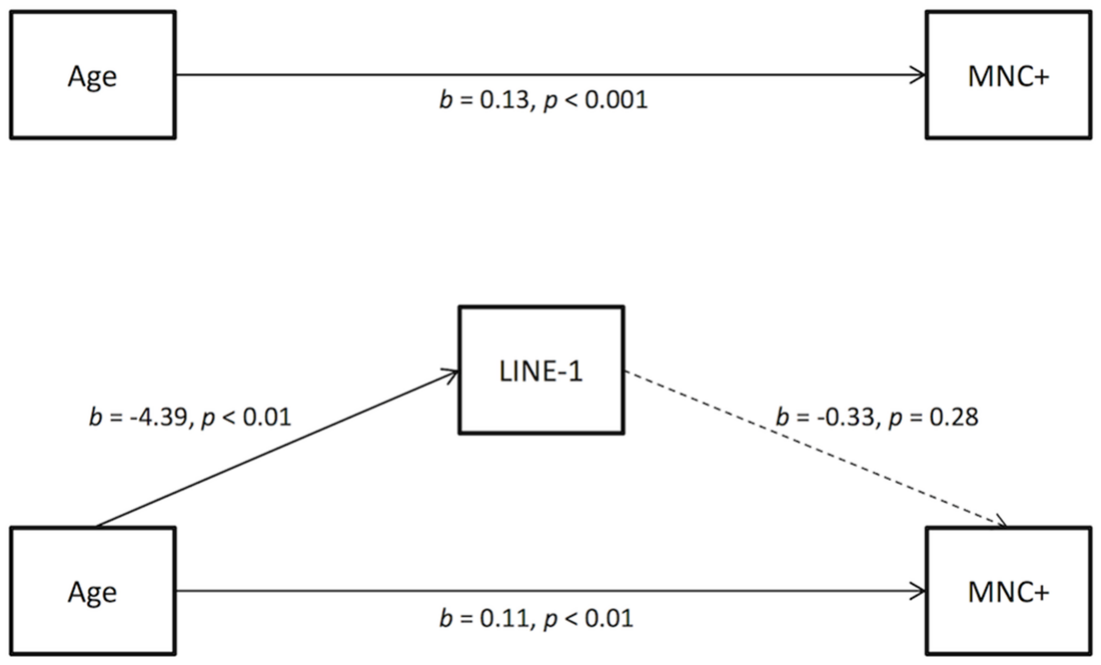
Causal model for age and LINE-1 hypomethylation prediction of MNC+. (Top) the path from age to MNC+. (Bottom) another path from age through LINE-1 hypomethylation to MNC+. Arrows connecting one variable to another represent unstandardized regression coefficients of the path. Solid lines are significant whereas dashed lines are not.

Fig 1 illustrates the causal path from age to MNC+ (top); and the mediational model of age, LINE-1 hypomethylation (the mediator), and MNC+ (bottom). As shown in the top panel of the figure, age significantly predicted MNC+, *b* = 0.13, *t*(30) = 4.51, *p* < 0.001, which is consistent with previous studies [29]. As shown in the bottom panel, age significantly predicted LINE-1 hypomethylation, *b* = -4.39, *t*(30) = -3.14, *p* < 0.01. More importantly, LINE-1 hypomethylation did not mediate the effect of age on MNC+ although it alone significantly predicted MNC+, *b* = -0.83, *t*(30) = -2.77, *p* < 0.01: that is, as shown in the bottom panel, there was no significant effect of age on MNC+ through LINE-1 hypomethylation, *b* = -0.33, *t*(30) = -1.11, *p* = 0.28 whereas there was a significant effect of age on MNC+ controlling for LINE-1 hypomethylation, *b* = 0.11, *t*(30) = 3.38, *p* < 0.01. In other words, when age and LINE-1 hypomethylation were used to predict MNC+, a total of 43% of the variance in MNC+ was significantly explained, *F*(2, 29) = 10.88, *p* < 0.001. However, only 3% of the total variance was explained by age through LINE-1 hypomethylation. The effect of age on MNC+ through LINE-1 hypomethylation was too small to be significant (Sobel’s *z* = 1.05, *p* = 0.29). To the best of our knowledge, this is the first time it has been shown that LINE-1 hypomethylation itself accounts for MNC+; however, in cases when age accounts for MNC+, the role of LINE-1 hypomethylation as a mediator is negligible.

## Discussion

The influence of aging on DNA methylation has been reported in various studies [8, 22, 24, 39–41]. The mapping of age related methylation changes in genomic DNA is important to completely understand the molecular basis of various diseases, including approximate age of onset. However, the reported results have not been consistent [6, 42], and the degree of hypomethylation that corresponds with age and certain exposure status is still unknown. In the present study, we examined the association between global genomic methylation and age, tobacco smoking, drinking status and MNC+, an age-dependent cytogenetic biomarker for genomic instability.

We measured methylation levels of the repetitive element, LINE-1 in WBC DNA known as a surrogate marker for global DNA methylation [43]. Recently, several studies reported that LINE-1 methylation could be used as an indicator for the influence of environmental conditions and life style habits on the genome [9–12, 44, 45]. Other data from several studies suggest that DNA methylation changes in WBC can serve as a useful biomarker for different health outcomes, although it is much more limited. Recently, a global decrease in the methylation of peripheral blood DNA was found to be an independent risk factor for many cancers [46]. Methylation patterns are known to be tissue specific, however, a recent study by Christensen et al. [47] showed that age-related global DNA hypomethylation appears to be similar across tissue types, suggesting that common mechanisms may underlie methylation changes over time.

The results obtained in this study demonstrate that LINE-1 methylation levels in WBC DNA is significantly decreased with increasing age among healthy male Korean subjects. Similarly, other reports have demonstrated that global DNA hypomethylation in the repetitive elements LINE-1 and/or Alu in WBC can change over time [8, 48]. The impact of aging on genomic DNA hypomethylation has also been reported using different assays that measure global DNA methylation levels [10, 17, 40]. In contrast, numerous studies have reported no age effect on blood LINE-1 methylation [6, 42, 49]. In addition, studies examining global methylation among cancer patients suggest no methylation changes occur during normal aging [7, 23]. This discordance within the literature may be partially explained by differences in race/ethnicity. In particular, Zhang et al. [6] reported significant differences in LINE-1 methylation by race/ethnicity, and most of the studies reporting no aging effects on global hypomethylation did not include Asian populations.

We also examined the association of LINE-1 methylation with MNC+ frequency, which is a well-known age-dependent cytogenetic biomarker. Our data showed that the repetitive element LINE-1 is significantly demethylated with increasing MNC+ frequency. MN in blood lymphocytes are a well-known cytogenetic biomarker, which represents a reliable test to assess chromosome damage and genomic instability that are induced by environmental exposures as well as aging [25–27]. MN are formed by whole chromosome loss or breaks and reflect genomic instability at the time of cell division [50]. In particular, increased MN frequency with age is mainly due to increased MNC+, a numerical chromosomal instability formed by whole chromosome loss [28, 29]. In this study, we used the micronucleus-centromere assay, which combines the CBMN assay with FISH technique. The micronucleus-centromere assay is a more sensitive method to detect age-dependent MN and MNC+ since it can determine whether MN are derived from acentric chromosome fragments or whole chromosomes [28, 51, 52].

Decreased global DNA methylation results in genomic instability and plays a critical role in the development of cancer and other diseases. Some specific biomarkers for genomic instability, such as MNC+, are age-dependent. Therefore, we further examined whether LINE-1 hypomethylation acts as an age-dependent contributor to MNC+, but the results were not statistically significant. This could be partially explained by the small number of individuals in our study, which precluded further statistical analysis for significance.

Tobacco smoking and alcohol consumption may be risk factors for global DNA hypomethyation. Indeed, some evidence supports a change in DNA methylation with smoking and alcohol intake [14, 16]. However, several other studies failed to find significant associations between genomic DNA hypomethylation and smoking [7, 23, 53, 54] or alcohol intake [6]. There were no significant associations between LINE-1 hypomethylation and smoking or alcohol in our study, but our results should be interpreted carefully due to small sample size.

The present study is an investigation of the associations between global hypomethylation, age and MNC+ formation. As described above, this study included a relatively small sample size of only 32 subjects, which somewhat limited the statistical power. The inclusion of only Korean males in our study population limits generalizability, since some studies have reported a significant decline in DNA methylation by gender and race [6, 55]. In addition, the lack of information regarding seasonality, a modifier of methylation levels in healthy populations [45], is also a limitation of this study. Despite these limitations, to our knowledge, this is the first study in healthy human volunteers to quantify the age-dependent global methylation levels along with a valuable age-dependent biomarker for genomic instability. Furthermore, this is also the first study to examine the role of LINE-1 hypomethylation in age-related MNC+ formation as a potential mechanism of age-dependent genomic instability.

## Conclusions

These studies confirm that repetitive element LINE-1 is significantly demethylated with increasing age and MNC+ frequency. However, there are no effects of cigarette smoking and alcohol intake on LINE-1 hypomethylation or MNC+. Further analysis to determine the role of LINE-1 methylation in age-related MNC+ showed that the relation between age and MNC+ was not significantly mediated by LINE-1 hypomethylation. Our results indicate that LINE-1 is demethylated with age and increasing MNC+, but additional studies with a larger number of subjects that includes both sexes and other repetitive elements using different assays are needed to fully understand the relationship among genomic DNA hypomethylation, age and genomic instability.

## Supporting Information

S1 Table Title: Age, MNC+ and LINE-1 data. Legend: “Data file Cho et al. xlsx” contains information on age, MNC+ and LINE-1 methylation, which were measured using the micronucleus-centromere and pyrosequencing assays as described in the paper. Also, the data file provides information on the smoking and drinking status of individuals used in the study.
(XLSX)
